# Bilateral temporal lobe dysplasia and seizure onset associated with biallelic *CNTNAP2* variants

**DOI:** 10.1002/epi4.12843

**Published:** 2023-12-15

**Authors:** Norman Panza, Claudia Bianchini, Valentina Cetica, Simona Balestrini, Carmen Barba, Anna Rita Ferrari, Davide Mei, Lucio Parmeggiani, Elena Parrini, Renzo Guerrini

**Affiliations:** ^1^ Neuroscience Department Meyer Children's Hospital IRCCS Florence Italy; ^2^ University of Florence Florence Italy; ^3^ IRCCS Stella Maris Calambrone, Pisa Italy; ^4^ Department of Pediatric Neurology Bolzano Hospital Bolzano Italy

**Keywords:** *CNTNAP2*, focal cortical dysplasia, genetic epilepsy, temporal lobe epilepsy

## Abstract

Biallelic *CNTNAP2* variants have been associated with Pitt‐Hopkins‐like syndrome. We describe six novel and one previously reported patients from six independent families and review the literature including 64 patients carrying biallelic *CNTNAP2* variants. Initial reports highlighted intractable focal seizures and the failure of epilepsy surgery in children, but subsequent reports did not expand on this aspect. In all our patients (n = 7), brain MRI showed bilateral temporal gray/white matter blurring with white matter high signal intensity, more obvious on the T2‐FLAIR sequences, consistent with bilateral temporal lobe dysplasia. All patients had focal seizures with temporal lobe onset and semiology, which were recorded on EEG in five, showing bilateral independent temporal onset in four. Epilepsy was responsive to anti‐seizure medications in two patients (2/7, 28.5%), and pharmaco‐resistant in five (5/7, 71.5%). Splice‐site variants identified in five patients (5/7, 71.5%) were the most common mutational finding. Our observation expands the phenotypic and genetic spectrum of biallelic *CNTNAP2* alterations focusing on the neuroimaging features and provides evidence for an elective bilateral anatomoelectroclinical involvement of the temporal lobes in the associated epilepsy, with relevant implications on clinical management.

## 1 INTRODUCTION

The *CNTNAP2* gene encodes the contactin‐associated protein‐like 2 (CASPR2), a neuronal transmembrane protein, member of the neurexin superfamily. CASPR2 is involved in neural‐glia interactions and in the clustering of potassium channels inside the juxtaparanodal regions of Ranvier nodes in myelinated axons^1^, ^2^ and plays a role in several neuronal processes such as neuronal migration, dendritic arborization, and spine development.^3^

The 24‐exons *CNTNAP2* gene spans 2.3‐Mb on chromosome 7q35. Being one of the largest genes in the human genome, covering nearly 1.5% of chromosome 7, *CNTNAP2* is prone to copy number variants (CNVs) and single nucleotide variants (SNVs).

Biallelic *CNTNAP2* variants have been associated with Pitt‐Hopkins‐like syndrome (MIM: #610042), a disorder characterized by developmental delay, intellectual disability, speech impairment or regression, behavioral abnormalities, and seizures.^4^ Malformation of cortical development has been histologically confirmed in three patients from a cohort of seven sibships belonging to two Old Order Amish families with a high rate of consanguinity.^5^ In a recent study, three out of 20 patients with biallelic *CNTNAP2* variants exhibited neuroimaging signal abnormalities consistent with focal cortical dysplasia (FCD) in the anterior temporal lobes.^6^ FCD has also been suspected in two additional patients from two unrelated families.^4^, ^7^

Here we describe seven patients harboring biallelic *CNTNAP2* variants, from six independent families, and demonstrate bilateral temporal lobe FCD with bilateral seizure onset as a prominent phenotypic feature associated with alterations in this gene.

## 2 METHODS

The study was approved by the Pediatric Ethics Committee of the Tuscany Region. Informed consent was obtained by patients, parents, or legal guardians.

We identified seven patients, from six unrelated families, carrying biallelic *CNTNAP2* pathogenic or likely pathogenic CNVs and SNVs from a cohort of about 7000 patients with neurodevelopmental disorders seen at the Meyer Children's Hospital IRCCS or referred from other hospitals for genetic testing in the last 8 years.

Six patients were studied with next generation‐sequencing (NGS) analysis of a panel of 220 epilepsy genes and one patient with whole‐exome sequencing (WES).

We reviewed the literature on *CNTNAP2* from March 2006 until April 2023 (Figure S1). Inclusion criteria were patients carrying biallelic likely pathogenic or pathogenic variants according to the American College of Medical Genetics and Genomics (ACMG) classification.^8^, ^9^ We identified 64 previously published patients and highlighted innovative anatomoelectroclinical features derived from our cohort.

Detailed methods are available in the Appendix S1.

## 3 RESULTS

We describe six novel patients and also included in the study one previously reported patient (Pt 2)^6^ with additional clinical data (Table S1).

### 3.1 Genetic features

In our cohort, four patients had homozygous splice‐site variants (Pts 1, 4a, 4b, 6), three patients were compound heterozygous for two deletions (Pt 2), or one gross deletion combined with a nonsense (Pt 5) or a splice‐site variant (Pt 3).

### 3.2 Clinical features

All patients (7/7; 100%) had global developmental delay, of mild (3/7; 43%), moderate (2/7; 28.5%), or severe (2/7; 28.5%) degree, associated with expressive language disturbance. A formal cognitive evaluation was available for four patients (4/7; 57%): two had normal intelligence quotient (IQ) scores (2/7; 28.5%), one had moderate (1/7; 14%), and one had mild intellectual disability (1/7; 14%).

Five patients (5/7; 71.5%) manifested behavioral disturbance. Patient 1 received a diagnosis of autism spectrum disorder (ASD). None of the patients received pharmacological treatment for the behavioral abnormalities.

Head circumference (HC) measures, at birth or at the last follow‐up, showed a trend for above average (>50th%) head size (Table S1).

### 3.3 Neuroimaging findings

**FIGURE 1:**
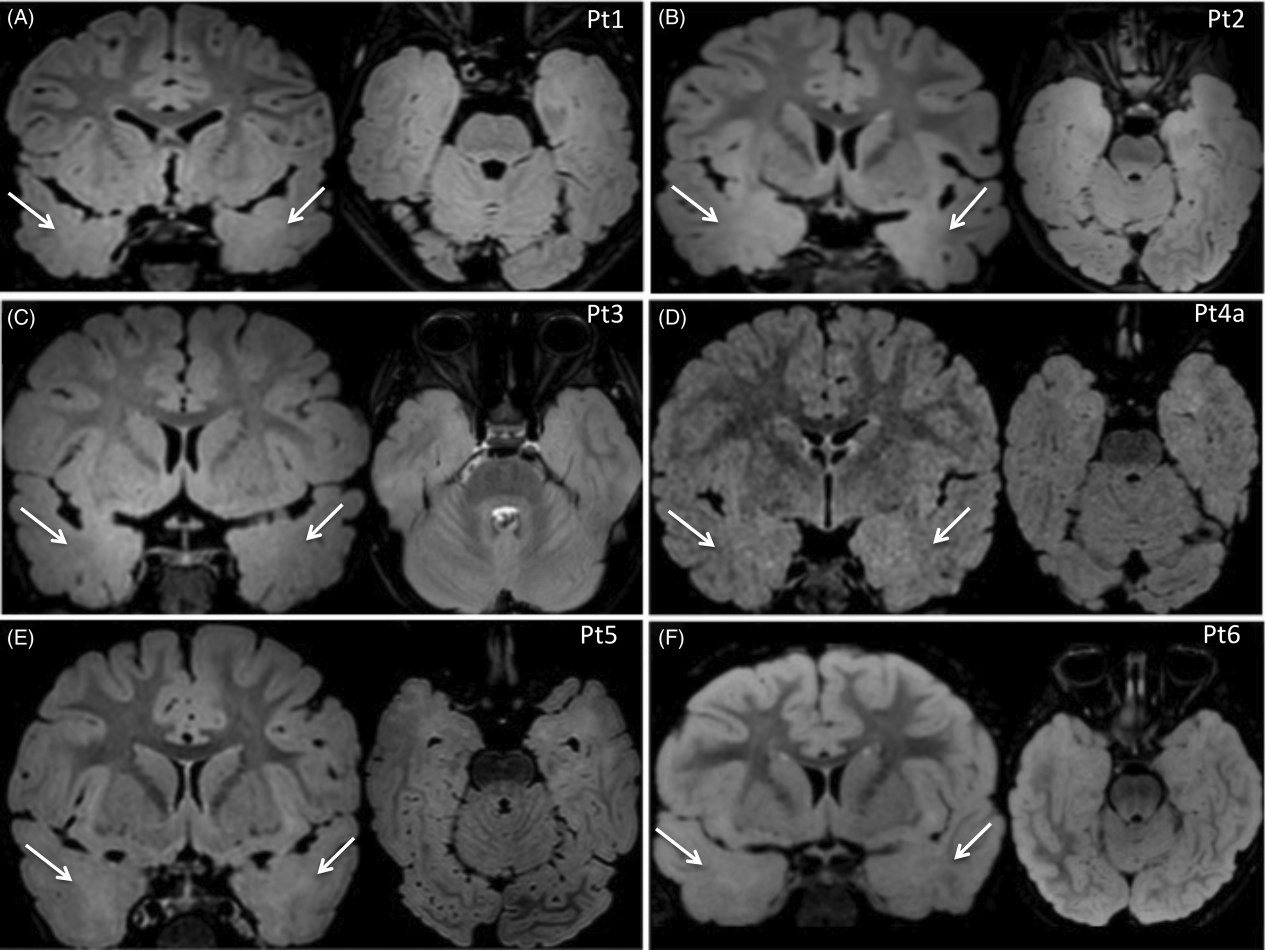
A, MRI of Patient 1 (3 y): T2‐weighted FLAIR coronal and axial sections show bilateral temporal gray/white matter (GM/WM) blurring (white arrows). B, MRI of Patient 2 (3 y): T2‐weighted FLAIR coronal and axial sections show bilateral temporal GM/WM blurring (white arrows). C, MRI of Patient 3 (9 y): T2‐weighted FLAIR coronal and axial sections show bilateral temporal GM/WM blurring (indicated by white arrows). D, MRI of Patient 4a (2 y): T2‐weighted FLAIR sections show coronal and axial bilateral temporal GM/WM blurring and WM signal hyperintensity (indicated by white arrows). E, MRI of Patient 5 (2 y): T2‐weighted FLAIR coronal and axial sections show GM/WM blurring and WM signal hyperintensity (white arrows). F, MRI of patient 6 (5 y): T2‐weighted FLAIR coronal and axial sections show bilateral temporal GM/WM blurring and WM signal hyperintensity (white arrows). All MRI images were performed at 3T.

Brain MRI was performed in all patients (7/7; 100%) and was reviewed by a multidisciplinary team. In all patients, images showed bilateral temporal cortical gray‐white matter blurring, with white matter high signal intensity, more obvious in the T2‐FLAIR axial and coronal sequences (Figure 1). The median age at which brain MRI was performed was 3 years. Patient 5 underwent two brain MRIs: the first, performed at age 1 year, revealed unilateral temporal involvement, whereas the second, 1 year later, revealed bilateral temporal involvement.

### 3.4 Epilepsy phenotype

All patients (7/7; 100%) had focal seizures originating from the temporal lobes, with a median age at onset of 1 year. Patient 3 (1/7; 14%) experienced recurrent febrile seizures between 11 months and 2 years of age. All patients were pharmacologically treated with different anti‐seizure medications (ASMs), used as mono‐ or polytherapy.

Seizure outcome in our patients was variable: Patient 2 was seizure‐free from age 4 till the last follow‐up at age 7, on an association of valproic acid (VPA) and lamotrigine (LTG); Patient 3 was seizure‐free from age 3 till the last follow‐up at age 6, on carbamazepine (CBZ); five patients were pharmaco‐resistant with ongoing seizures on a weekly (Pt 1, 4a, 4b) or monthly (Pt 5, 6) basis with age at last follow‐up ranging from 3 to 19 years. CBZ was effective or transiently effective in four patients (4/7; 57%).

**FIGURE 2:**
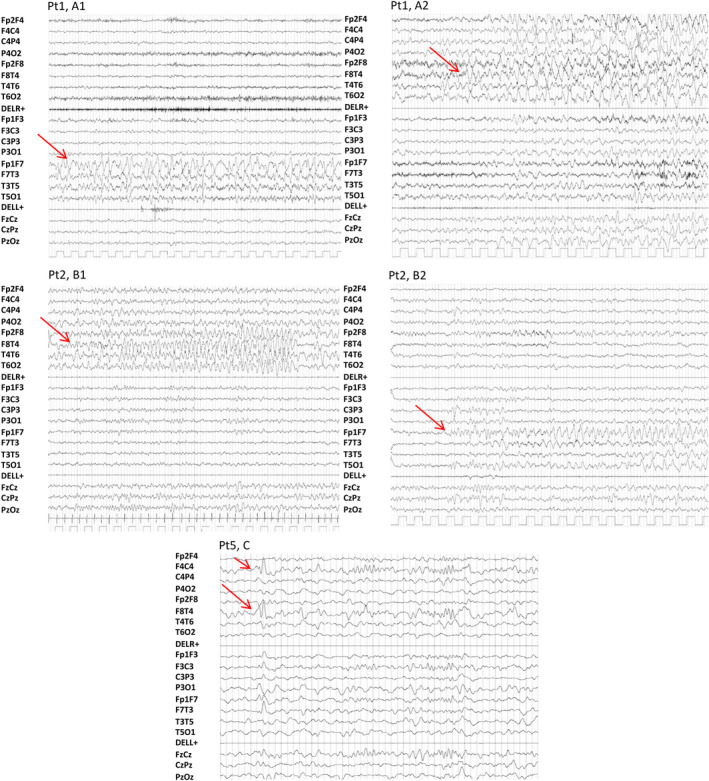
Ictal and interictal EEG of Patients 1, 2, and 5 are indicated by red arrows. A, Ictal EEG patterns of Patient 1, showing independent left (A1) and right (A2) ictal discharges. B, Ictal EEG pattern of Patient 2, showing independent right fronto‐temporal (B1) and left frontal (B2) seizure activity. C, Interictal EEG patterns of Patient 5, showing right fronto‐temporal sharp waves.

All patients (7/7; 100%) had focal interictal EEG discharges involving the temporal regions, which were unilateral in two patients (2/7; 28.5%), and bilateral in five (5/7; 71.5%). In five patients (5/7; 71.5%), seizures with temporal lobe origin were video‐EEG recorded during wakefulness and sleep; in four of them (4/5; 80%) seizures originated independently from either side (Figure 2). Ictal semiology was characterized by gestural and oral automatisms, and impaired awareness, followed by tonic asymmetric posturing.

From the literature, we identified 64 patients harboring 36 different pathogenic or likely pathogenic biallelic *CNTNAP2* variants (Figure S2), whose neuroimaging, epilepsy, and molecular genetic details are summarized in Table S2.

## 4 DISCUSSION

*CNTNAP2* has a crucial role in neuronal development and has been associated with a broad spectrum of neuropsychiatric disorders. Our study expands the phenotypic and genetic spectrum of biallelic *CNTNAP2* alterations providing clear evidence for an elective bilateral anatomoelectroclinical involvement of the temporal lobes in the associated epilepsy, with relevant implications on clinical management.

### 4.1 Genetic features

Combining previously published patients (n = 64) and our cohort (n = 7), most biallelic *CNTNAP2* pathogenic variants were nonsense, frameshift, or gross deletions/duplications (Tables S1 and S2). Splice‐site variants were previously described only in three patients^6^, ^7^, ^10^ (3/64; 5%) but account for five patients in our cohort (5/7; 71.5%). All splice‐site variants were homozygous in our patients, except for the c.550 + 1G>T that co‐occurred with a deletion of exons 4–5. The variant c.1084‐2A>G identified in two patients (Pt 4a, 4b), was predicted to alter the splice acceptor site of exon 8, and is in a very conserved site with a Combined Annotation Dependent Depletion (CADD) score of 35. Both patients share the same ancestors and live in a small, isolated valley in the Italian region of South Tyrol. The population‐based haplotype analysis of the South Tyrol population, performed in the context of the Cooperative Health Research In South Tyrol (CHRIS) initiative,^11^ identified an enrichment of heterozygous carriers of this variant (c.1084‐2A>G), likely indicating a founder effect favored by the geographic isolation of this valley.

The clinical presentation of patients harboring biallelic SNVs, biallelic CNVs, or a combination of one SNV and one CNV was indistinguishable, suggesting that there is no evident genotype–phenotype correlation based on the type of biallelic *CNTNAP2* alterations.

### 4.2 Clinical features

Previously described patients with likely pathogenic and pathogenic biallelic variants in *CNTNAP2* exhibited global developmental delay, intellectual disability, speech and behavioral abnormalities, seizures, and, occasionally, MRI abnormalities.

The seven patients in our cohort exhibit a typical *CNTNAP2* core phenotype, including global developmental delay with expressive language disturbances in all (7/7, 100%) and behavioral abnormalities in most (5/7, 71%). In addition, neuroimaging and epilepsy findings in our cohort illustrate some innovative features.

### 4.3 Neuroimaging findings

All our patients (7/7; 100%) showed cortical dysplasia‐like MRI findings, which were more prominent in T2‐FLAIR sequences. Out of 44 previously reported patients harboring pathogenic or likely pathogenic biallelic *CNTNAP2* variants whose MRI findings were described, seven (7/44; 16%) exhibited similar cortical dysplasia‐like findings on neuroimaging.^4^, ^5^, ^6^, ^7^ It is possible that visible, yet subtle, MRI changes consistent with FCD might have been overlooked in other patients with *CNTNAP2* variants, as imaging suspicion of FCD is often based on unilateral or asymmetric changes, whilst bilateral symmetric T2‐ FLAIR signal hyperintensity in the limbic and paralimbic cortex can be artifactual in healthy controls.^12^ Four previously reported patients (4/64; 6%) underwent epilepsy surgery but experienced recurrence of seizures 5 to 15 months after the operation.^5^, ^7^ In three of them, histopathology confirmed malformation of cortical development and showed abnormally‐organized neuronal and glia cells and increased cellular density in brain specimens throughout the hippocampus, amygdala, neocortex, and subcortex,^5^ corroborating the hypothesis that the cerebral anomalies accompanying *CNTNAP2* biallelic variants are widespread as also in part suggested by the increased head size (above average) of patients. A trend for above‐average head size was also present in our patients (Table S1).

### 4.4 Epilepsy phenotype

Focal seizures arising from the temporal regions, consistent with previous observations,^4^, ^5^, ^6^ were observed in all our patients (7/7; 100%). Epilepsy severity was variable, with two patients (2/7; 28.5%) being seizure‐free on ASMs, and five pharmaco‐resistant (5/7; 71.5%). We observed that seizures independently originated from either temporal lobe in four patients (4/7; 57%), a finding that confirms an underlying nonlocalized structural abnormality but highlights how temporal lobe epileptogenesis is prominent in this genetic disorder.

Temporal lobe seizure onset and semiology may be misleading and prompt a hypothesis of temporal lobectomy if bilateral distribution of brain abnormalities is not fully appreciated and bilateral independent temporal seizure onset is not captured by recording an adequate number of seizures. It is unclear whether the surgical failures reported by Strauss et al. (2016) and Sanders et al. (2019) in all four patients who underwent temporal lobectomy were related to contralateral or more widespread seizure onset,^5^, ^7^ but our findings suggest this possibility.

Our results provide evidence of a bilateral independent anatomoelectroclinical involvement of the temporal lobes in *CNTNAP2*‐associated epilepsy, which negatively affects surgical treatment options and the overall epilepsy outcome.

Identification of a genetic etiology can impact treatment choice in epilepsies, in terms of selecting or avoiding specific treatment choices, for example, sodium channel blockers are recommended in epilepsies caused by gain‐of‐function variants in sodium channel genes but should be avoided in Dravet syndrome, caused by *SCN1A* loss‐of‐function variants.^13^ Our findings provide a further example of management strategies driven by genetic diagnosis, that is, surgical treatment would seem to be unsuccessful in patients with *CNTNAP2*‐related epilepsy. This observation is in line with a literature review of seizure outcomes following epilepsy surgery in patients with different genetic causes of refractory epilepsy which suggested the ineffectiveness of surgery in patients with variants in genes involved in synaptic transmission.^14^ Although the number of observations remains limited, a note of caution towards any surgical approach appears to be appropriate based on available information. We also highlight the importance of careful review of “negative” brain MRI scans, in light of novel genetic findings and electroclinical features. Genetic testing should be routinely integrated into the presurgical evaluation of patients with refractory focal epilepsy to drive a more “precise” selection of surgical candidates.

## CONFLICT OF INTEREST STATEMENT

The authors have no conflict of interest to disclose. The authors confirm that they have read the Journal's position on issues involved in ethical publication and affirm that this report is consistent with those guidelines.

## ETHICAL APPROVAL

The study was approved by the Pediatric Ethics Committee of the Tuscany Region and informed consent was obtained by patients, parents, or legal guardians.

## ETHICAL PUBLICATION STATEMENT

We confirm that we have read the Journal's position on issues involved in ethical publication and affirm that this report is consistent with those guidelines.

## Supporting information


Appendix S1

Appendix S2

## Data Availability

The data that support the findings of this study are available from the corresponding author upon reasonable request.
