# Supplementary material for: Bilateral temporal lobe dysplasia and seizure onset associated with biallelic CNTNAP2 variants
Source: Epilepsia Open. 2023 Dec 15;9(1):417–23. doi: 10.1002/epi4.12843 (PMC10839365; doi:10.1002/epi4.12843)
Supplement: Supplementary file 1 — Appendix S1 [file EPI4-9-417-s002.doc]

**Supplementary methods:**

All patients underwent prolonged scalp video-EEG monitoring and structural 1.5 or 3T brain MRI. Brain MRI protocols included standard diagnostic sequences such as 3D T2-FLAIR, T2 Gradient Echo (GRE), T2-Fast Spin Echo (FSE), T1-Spin Echo (S)E and 3D T1-weighted fast-spoiled gradient echo (FSPGR) images of the whole brain.

Four patients (4/7, 57%) underwent a formal neuropsychological assessment with Leiter, WPSSI-III or Griffiths scales.^1^

Genetic analysis was performed at the Meyer Children's Hospital IRCCS. Six patients underwent next generation sequencing (NGS) analysis of a panel of 220 epilepsy genes and one patient whole exome sequencing (WES).

In brief, the DNA extracted from peripheral blood leukocytes using a QiaSymphony SP robot (Qiagen) was quantified with a Quantifluor Fluorometer (Promega) and used to perform a target enrichment and library preparation with a custom designed SureSelect QXT panel (Agilent) including 220 genes associated with epilepsy in six patients and a whole-exome sequencing in one patient (SureSelect XT2 Clinical Research Exome, Agilent). The obtained libraries were then sequenced on a NextSeq 550 instrument (Illumina, USA) using a 2x150bp paired end protocol.

Reads were aligned to human genome build GRCh37/UCSC hg19 and variants called according to GATK’s best practices, annotated with VarSeq (Golden Helix), and filtered against public (gnomAD V.2.0) and *in-house* databases, to retain private (present in only one patient) and rare (minor allele frequency (MAF) < 0.1%) exonic and splice-site variants. Variants with depth >10 and genotype quality >20 were then filtered according to the *de novo* autosomal dominant, X-linked, homozygous recessive, and compound heterozygous models. We evaluated the functional impact of variants through *in-silico* prediction using the dbNSFP database (v3.3a).^2^ We analyzed all splice-site variants using the splice-site prediction tools available on the Alamut Visual platform (https://www.interactive-biosoftware.com/alamut-visual/). We obtained variants validation with Sanger sequencing on both strands, using the Big Dye Terminator V3.1 chemistry on a 3500 DX Genetic Analyzer (Thermo Fisher Scientific).

Copy number variants (CNVs) analysis was obtained with VarSeq CNV calling algorithm relies on coverage information computed from BAM files. CNVs validation was performed by Array-CGH using the 180K Agilent platform (Agilent Technologies, Santa Clara, CA, USA) and the Genomic Workbench software, which provides a practical average resolution of 40Kb, or SYBR Green-based real-time PCR (Power SYBR™ Green PCR Master Mix, Applied Biosystems™) using custom designed primers.

We filtered CNVs against the Database of Genomic Variants (DGV; http://dgv.tcag.ca/) and checked overlapping of selected CNVs with the DatabasE of genomiC variation and Phenotype in Humans using Ensembl Resources (DECIPHER, https://decipher.sanger.ac.uk).^3^ We classified variants and CNVs according to the international guidelines of the American College of Medical Genetics and Genomics (ACMG) Laboratory Practice Committee Working Group.^4,5^ We reviewed the literature for previously published patients carrying biallelic *CNTNAP2* pathogenic or likely pathogenic variants. Inclusion criteria were biallelic genetic alterations including CNVs (i.e., gross deletions or duplications involving the *CNTNAP2* gene), and missense, nonsense and frameshift variants, classified as pathogenic or likely pathogenic according to the ACMG guidelines.

We excluded those patients harboring variants of uncertain significance or carrying one single heterozygous variant, even if classified as pathogenic or likely pathogenic.

In the PubMed database, we searched for all publications including *CNTNAP2* variants by using as MeSH terms “*CNTNAP2*” and either “mutations” or “variants”, within the time frame ranging from the first published patient (Verkerk AJ et al. 2003 – patient excluded because carrying a single heterozygous *CNTNAP2* variant^6^) until April 2023. The reference lists of the selected publications were checked for further potentially suitable publications. The pathogenic variants identified in the selected publications were cross checked with the *CNTNAP2* variants listed in the Human Gene Mutation Database and data on the associated neurological phenotype were extracted, when available (Figure S1).

We included 64 previously reported patients harboring 36 different biallelic *CNTNAP2* pathogenic or likely pathogenic CNVs and SNVs.

**Supplementary tables**

**Table S1**

| **ID Patient** | **1** | **2** | **3** | **4a (second-degree cousin of Pt 4b)** | **4b (second-degree cousin of Pt 4a)** | **5** | **6** |
| --- | --- | --- | --- | --- | --- | --- | --- |
| **Gender** | M | M | M | F | F | M | F |
| **Age at first medical evaluation** | 2y | 2y | 3y | 1y | NA | 1y6m | 1y2m |
| ***CNTNAP2* variants (NM_014141.6)** | c.1777+2T>^7^ | deletion exons 2-3^8^, pat | c.550+1G>T, pat | c.1084-2A>G | c.1084-2A>G | c.3283C>T, p.(Arg1095^*^),^9^ pat | c.2555-1G>C |
|  | c.1777+2T>^7^ | deletion exons 2-8^8^, mat | deletion exons 4-5, mat | c.1084-2A>G | c.1084-2A>G | deletion exon 3,^10^ mat | c.2555-1G>C |
| **ACMG classificatio^4,5^** | P | LP/LP | P/LP | P | P | P/LP | P |
| **ACMG criteria^4,5^** | PVS1, PM2 | 2B, 2E/2B, 2E | PVS1, PM2/2B, 2E | PVS1, PM2 | PVS1, PM2 | PVS1, PM2, PP5/2B, 2E | PVS1, PM2 |
| **Age and overall clinical condition at last follow-up** | 10y/DD, FoS, strabismus, scoliosis | 7y/DD, FoS, BA | 6y/DD, FeS (11m-2y), FoS | 5y/DD, FoS, BA | 19y/DD, FoS, BA | 3y/DD, FoS, BA | 9y/DD, FoS, BA, no language |
| **HC at birth (%)/HC last available (%, age in y)** | 34 cm (35th %)/49 cm (50th%, 5y) | 35 cm (50th%)/52 cm (75th%, 5y) | 35 cm (50th%)/49 cm (50th%, 5y) | 35 cm (85th%)/NA | 35 cm (85th%)/NA | 35 cm (50th%)/ 49 cm (50th%), 2y6m) | NA |
| **Early developmental delay** | Moderate-severe | Mild | Mild | Mild | Moderate | Moderate | Moderate-severe |
| **Cognitive level (age in y)** | DQ<50 (2y Griffiths scale) | IQ 76 (5y Leiter scale) | IQ 83 (3y10m WPSSI-III) | NA | NA | DQ 55 (2y Griffiths scale) | NA |
| **Age at sz onset/type(s)** | 1y/FoS | 2y/FIAS | 0.9y/FeS | 1y/FoS | 1y/NA | 1y/FIAS | 1y/FoS |
|  |  |  | 2y/FoS |  |  |  |  |
| **Epilepsy syndrome** | Focal epilepsy | Focal epilepsy | Febrile szs and focal epilepsy | Focal epilepsy | Focal epilepsy | Focal epilepsy | Focal epilepsy |
| **Epilepsy outcome at last follow-up** | Weekly szs | Sz free since age 4y (on VPA and LTG) | Sz free since 3y6m (on CBZ) | Weekly szs (recurrent clusters) | Weekly szs | Monthly szs | Monthly szs |
| **ASMs (ever tried)** | ACTH(-), ZNS(-), TPM(-), PHT(-), RUF(-) | CBZ(-), PHT(-), CLB(-), LCS(-), VPA(+), LTG(+) | VPA(-), TPM(-), CBZ(+) | CBZ(+-), OXC(-), PHT(-), LEV(-), TPM(-), LCS(-), CLB(-) | VPA(-), OXC(-), PHT(-), TPM(-), LCS(-), RUF(-), ZNS(-), LEV(-), PER | LEV(-), CLN(-), CBZ(+-), PHT(+) | CBZ(+-) TPM(+-) |
| **Interictal EEG/ictal onset** | Multifocal bilateral spikes and spike-waves/Independent R and L temporal onset | Multifocal bilateral spikes/ Independent R and L temporal lobe onset | R temporal spikes and sharp-waves/NA | Bilateral independent temporal spikes/Independent R and L temporal lobe onset | R temporal spike-waves/Independent R and L temporal lobe onset | R temporal theta waves/R fronto-temporal ictal onset | Bilateral fronto-temporal (R>L) slow waves /NA |
| **Age/Brain MRI** | 9y/Bilateral temporal GM/WM blurring (T2-FLAIR) | 3y/Bilateral temporal GM/WM blurring (T2-FLAIR) | 3y/Bilateral temporal, GM/WM blurring (T2-FLAIR) | 2y/Bilateral (L>R) mesial-temporal GM/WM blurring and bilateral temporal WM hyperintensity (T2-FLAIR) | NA/Bilateral temporal GM/WM blurring | 2y/Bilateral temporal GM/WM blurring and bilateral temporal WM hyperintensity (T2-FLAIR) | 5y/Bilateral (R>L) temporal, GM/WM blurring and bilateral temporal WM hyperintensity (T2-FLAIR) |

**Table S1**. Clinical features and molecular findings of our cohort. Legend: ACMG: American College of Medical Genetics; ACTH: adrenocorticotropic hormone; ASMs: anti-seizure medications; BA: behavioral abnormalities; CBZ: carbamazepine; CLB: clobazam; CLN: clonazepam; DD: developmental delay; DQ: developmental quotient; F: female; FeS: febrile seizures; FIAS: focal impaired awareness seizures; FLAIR: fluid-attenuated-inversion recovery; FoS: focal seizures; GM: grey matter; HC: head circumference; IQ: intelligence quotient; L: left; LCS: lacosamide; LEV: levetiracetam; LP: likely pathogenic; LTG: lamotrigine; M: male; m: months; mat: maternal; MD: movement disorder; MRI: magnetic resonance imaging; NA: not available; OXC: oxcarbazepine; P: pathogenic; pat: paternal; PER: perampanel; PHT: phenytoin; PM: pathogenic, moderate; PP: pathogenic, supporting; Pt: patient; PVS: pathogenic, very strong; R: right; RUF: rufinamide; sz(s): seizure(s); TPM: topiramate, VPA: valproic acid, WM: white matter; WPSSI-III: Wechsler preschool and primary scale of intelligence; y: years; ZNS: zonisamide; +: efficacy; -: lack of efficacy; +-: partial efficacy.

**Supplementary figures**

**Figure S1**


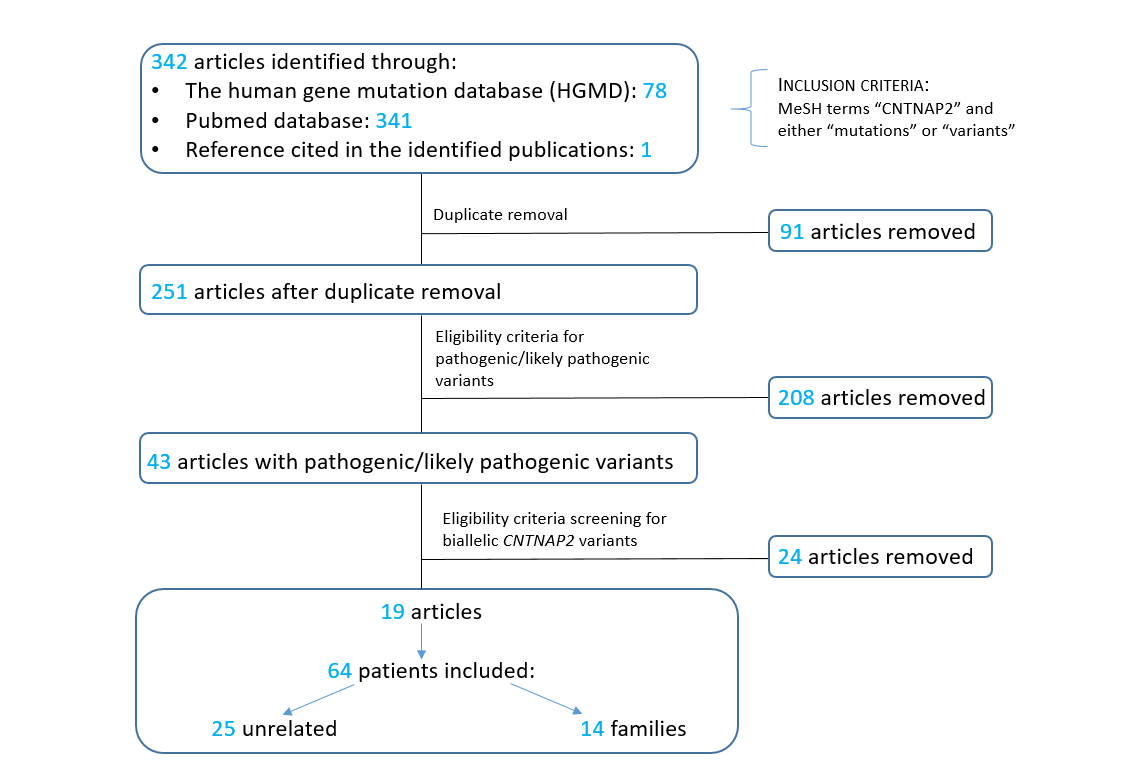


**Figure S1.** Flow diagram illustrating the stepwise approach used for inclusion of the relevant articles in the literature review.

**Figure S2**

**
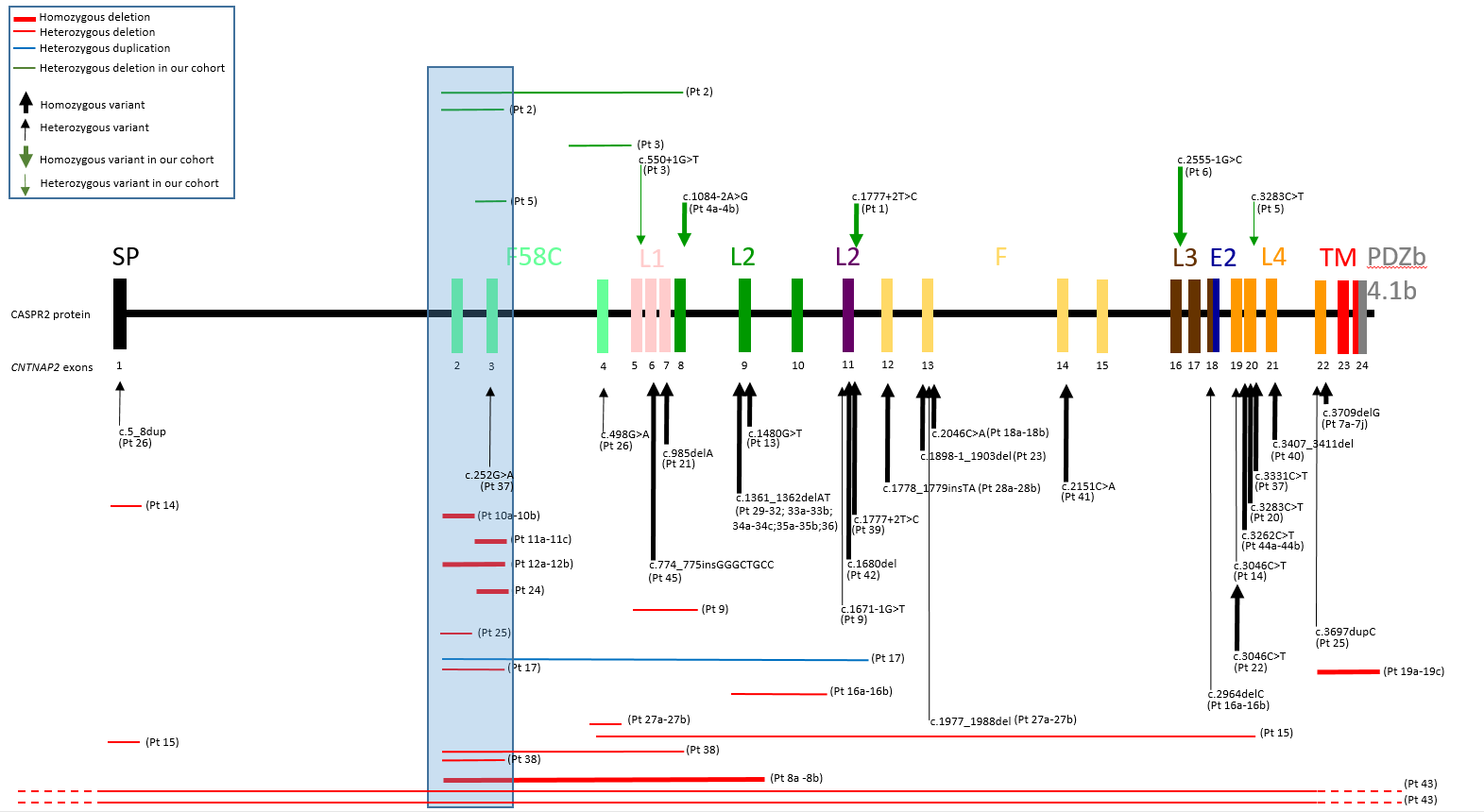
**

**Figure S2**. Distribution of *CNTNAP2* single nucleotide variants (SNVs) and copy number variants (CNVs) in exons and in the corresponding protein domains of CASPR2 identified in patients reported in the literature (in the lower part) and in our cohort (in the upper part). Patient numbers are referred to Table S1 and Table S2. In the light blue rectangle, the region more prone to CNVs includes exons 2-3 of the *CNTNAP2* gene. Legend: 4.1Bb: 4.1B-binding domain (grey); E1: epidermal growth factor-like domain 1 (purple), E2: epidermal growth factor-like domain 2 (blue); F: fibrinogen (yellow), F58C: discoidin-like domain (light green); L1: laminin G-like domain 1 (pink); L2: laminin G-like domain 2 (green); L3: laminin G-like domain 3 (brown); L4: laminin G-like domain 4 (orange); PDZb: PDZ-binding domain (grey).Pt: patient; SP: signal peptide (black);TM: transmembrane domain (red).

**Supplementary references**

1. Jayakar P, Gaillard WD, Tripathi M, Libenson MH, Mathern GW, Cross JH. Task Force for Paediatric Epilepsy Surgery, Commission for Paediatrics, and the Diagnostic Commission of the International League Against Epilepsy. Diagnostic test utilization in evaluation for resective epilepsy surgery in children. Epilepsia. 2014 Apr;55(4):507-18. doi: 10.1111/epi.12544. Epub 2014 Feb 11. PMID: 24512473.
2. Liu X, Wu C, Li C, Boerwinkle E. dbNSFP v3.0: A One-Stop Database of Functional Predictions and Annotations for Human Nonsynonymous and Splice-Site SNVs. Hum Mutat. 2016 Mar;37(3):235-41. doi: 10.1002/humu.22932. Epub 2016 Jan 5. PMID: 26555599.
3. Firth H.V., Shola M. Richards, A. Paul Bevan, Stephen Clayton, Manuel Corpas, et al. DECIPHER: Database of Chromosomal Imbalance and Phenotype in Humans Using Ensembl Resources, Am J Hum Genet. 2009 Apr;84(4):524-33. doi: 10.1016/j.ajhg.2009.03.010. Epub 2009 Apr 2. PMID: 19344873.
4. Richards S., Aziz N., Bale S., Bick D., Das S., Gastier-Foster J.,et al. (2015). Standards and guidelines for the interpretation of sequence variants: A joint consensus recommendation of the American College of Medical Genetics and Genomics and the Association for Molecular Pathology. Genetics in Medicine, 2015 May;17(5):405-2417, doi: 10.1038/gim.2015.30. PMID: 25741868.
5. Riggs ER, Andersen EF, Cherry AM, Kantarci S, Kearney H, Patel A, et al. Technical standards for the interpretation and reporting of constitutional copy-number variants: a joint consensus recommendation of the American College of Medical Genetics and Genomics (ACMG) and the Clinical Genome Resource (ClinGen). Genet Med. 2020 Feb;22(2):245-257. doi: 10.1038/s41436-019-0686-8. Epub 2019 Nov 6. Erratum in: Genet Med. 2021 Nov;23(11):2230. PMID: 31690835.
6. Verkerk AJ, Mathews CA, Joosse M, Eussen BH, Heutink P, Oostra BA. Tourette Syndrome Association International Consortium for Genetics. *CNTNAP2* is disrupted in a family with Gilles de la Tourette syndrome and obsessive compulsive disorder. Genomics. 2003 Jul;82(1):1-9. doi: 10.1016/s0888-7543(03)00097-1. PMID: 12809671.
7. Parrini E, Marini C, Mei D, Galuppi A, Cellini E, Pucatti D, et al. Diagnostic Targeted Resequencing in 349 Patients with Drug-Resistant Pediatric Epilepsies Identifies Causative Mutations in 30 Different Genes. Hum Mutat. 2017 Feb;38(2):216-225. doi: 10.1002/humu.23149. Epub 2016 Dec 9. PMID: 27864847.
8. D'Onofrio G, Accogli A, Severino M, Caliskan H, Kokotović T, Blazekovic A, et al. Genotype-phenotype correlation in contactin-associated protein-like 2 (CNTNAP-2) developmental disorder. Hum Genet. 2023. doi: 10.1007/s00439-023-02552-2. PMID: 37183190.
9. Shokoohi M, Hajjari M, Mohammadiasl J, Birgani MT. Whole Exome Sequencing found a Novel Truncating Mutation within CNTNAP2 Gene in an Iranian Patient with Mental Retardation. J Genet Disor Genet Rep 7:3. 2018. doi: 10.4172/2327-5790.1000177 (no pubmed ID).
10. Smogavec M, Cleall A, Hoyer J, Lederer D, Nassogne MC, Palmer EE,et al. Eight further individuals with intellectual disability and epilepsy carrying bi-allelic *CNTNAP2* aberrations allow delineation of the mutational and phenotypic spectrum. J Med Genet. 2016 Dec;53(12):820-827. doi: 10.1136/jmedgenet-2016-103880. Epub 2016 Jul 20. PMID: 27439707.
